# Letter to the editor - round table unites to tackle culture change in an effort to improve animal research reporting

**DOI:** 10.1186/s12917-017-1235-9

**Published:** 2017-11-07

**Authors:** Nicola J. Osborne, Merel Ritskes-Hoitinga, Amrita Ahluwahlia, Sabina Alam, Matthew Brown, Hayley Henderson, Wim de Leeuw, Joan Marsh, David Moher, Erica van Oort, Frances Rawle, Beat M. Riederer, Jose Sanchez-Morgado, Emily S. Sena, Caroline Struthers, Matthew Westmore, Marc T. Avey, Rony Kalman, Annette O’Connor, Jan Sargeant, Anja Petrie, Adrian Smith

**Affiliations:** 1 Responsible Research in Practice, Bailey House, 4-10 Barttelot Road, Horsham, West Sussex RH12 1DQ UK; 2 Radboud University Medical Centre, PO Box 9101, ML-6500 HB Nijmegen, The Netherlands; 3 Barts & The London School of Medicine & Dentistry, Queen Mary University of London, Charterhouse Square, London, EC1M 6BQ UK; 4 Faculty of 1000, Middlesex House, 34–42 Cleveland St, London, W1T 4LB UK; 5 Wellcome Trust, Gibbs Building, 215 Euston Road, London, NW1 2BE UK; 6 BMC Veterinary Research, BioMed Central, 236 Grays Inn Road, London, WC1X 8HB UK; 7 Animal Welfare Body Utrecht, Nieuw Gildestein, Room 1.81, Box 80125, NL - 3508 TC Utrecht Utrecht, The Netherlands; 8 kamer 1.82, PO Box 12007, 3501 AA Utrecht, The Netherlands; 9 The Lancet Psychiatry, 125 London Wall, London, EC2Y 5AS UK; 10 Centre for Journalology, Clinical Epidemiology Program, Ottawa Hospital Research Institute, The Ottawa Hospital - General Campus, 501 Smyth Rd, Room L1288, Ottawa, ON K1H 8L6 Canada; 11 ZonMw, PO Box 93 245, 2509 AE The Hague, The Netherlands; 12 Medical Research Council, 13th floor, One Kemble Street, London, WC2B 4AN UK; 13 Platform of Morphology, Faculty of Biology and Medicine, University of Lausanne, Rue du Bugnon 9, CH-1005 Lausanne, Switzerland; 14 Centre for Psychiatric Neuroscience, Department of Psychiatry, University Hospital of the Canton Vaud, route de Prilly, CH-1008 Lausanne-Prilly, Switzerland; 15 Trinity College Dublin, The University of Dublin, College Green, Dublin 2, Ireland; 16 Centre for Clinical Brain Sciences, University of Edinburgh, Chancellor’s Building, 49 Little France Crescent, Edinburgh, EH16 4SB UK; 17 Centre for Statistics in Medicine, Nuffield Dept. of Orthopaedics, Rheumatology and Musculoskeletal Sciences (NDORMS), University of Oxford, Botnar Research Centre, Nuffield Orthopaedic Centre, Windmill Road, Oxford, OX3 7LD UK; 18 National Institute for Health Research Evaluation, Trials and Studies Coordinating Centre, University of Southampton, Alpha House, Enterprise Road, Southampton, SO16 7NS UK; 19 ICF, 2635 Meridian Parkway Suite 200, Durham, NC 27713 USA; 20 Authority for Biological and Biomedical Models, The Hebrew University of Jerusalem, Ein Kerem, 91120 Jerusalém, Israel; 21 Lloyd Vet Med Center Rm 2424, College of Veterinary Medicine, Iowa State University, 1809 S Riverside Drive, Ames, IA 50011-3619 USA; 22 Centre for Public Health and Zoonoses, and Department of Population Medicine, 2502 Stewart Building, Ontario Veterinary College, University of Guelph, Guelph, ON N1G 2W1 Canada; 23 The University of Aberdeen, Medical Research Facility, Aberdeen, AB25 2ZD UK; 24 Norecopa, c/o Norwegian Veterinary Institute, P.O. Box 750 Sentrum, 0106 Oslo, Norway

## Abstract

A round table discussion was held during the LAVA-ESLAV-ECLAM conference on Reproducibility of Animal Studies on the 25th of September 2017 in Edinburgh. The aim of the round table was to discuss how to enhance the rate at which the quality of reporting animal research can be improved. This signed statement acknowledges the efforts that participant organizations have made towards improving the reporting of animal studies and confirms an ongoing commitment to drive further improvements, calling upon both academics and laboratory animal veterinarians to help make this cultural change.

## Main text

On Monday 25th September 2017 a round table meeting funded by LAL was held to discuss how to enhance the rate at which the quality of reporting animal research can be improved. This meeting took place during the LAVA-ESLAV-ECLAM conference on reproducibility in animal studies in Edinburgh. It was attended by individuals & organisations proactively working on this issue across Europe including research funders (MRC, Wellcome Trust, ZonMw), journals (EMBO, F1000Research, PLOS ONE, BMJ Open Science, BMC, British Journal of Pharmacology (BJP), The Lancet, Laboratory Animals) & others including ESLAV, EQUATOR, CAMARADES, SYRCLE, Responsible Research in Practice. NIHR were also present to share learning from the perspective of a funder of later phase clinical research.

All reiterated a commitment to drive improvements in the quality of animal research reporting to improve reproducibility, and call upon both academics and laboratory animal veterinarians to help drive this cultural change. In our experience relevant and useful training in experimental design, planning, reporting and data sharing is not universal within academic institutions, despite the perception that it might be. This must change, and academic institutions need to act now if scientists are to be skilled in the conduct of reproducible and reliable animal studies.

Yours Faithfully.

Round table participants: Nicola J Osborne^*^ (Responsible Research in Practice & SYRCLE ambassador), Merel Ritskes-Hoitinga^*^ (SYRCLE & ECLAM), Amrita Ahluwalia (British Journal of Pharmacology), Sabina Alam (F1000Research), Matthew Brown (Wellcome Trust), Hayley Henderson (BMC Veterinary Research), Wim de Leeuw (University of Utrecht), Joan Marsh (The Lancet & EASE), David Moher (Centre for Journalology, Ottawa Hospital Research Institute), Erica van Oort (ZonMw), Frances Rawle (Medical Research Council), Beat Riederer (Laboratory Animals), Jose Sanchez-Morgado (ESLAV), Emily Sena (CAMARADES & BMJ Open Science), Caroline Struthers (EQUATOR), Matt Westmore (National Institutes for Health Research).

^*^ Co-organisers of the round table meeting.

Supporters: Marc Avey (Canadian CAMARADES group & SYRCLE ambassador), Rony Kalman (ECLAM), Annette O’Connor and Jan Sargeant (MERIDIAN), Anja Petrie (LAVA), Adrian Smith (Norecopa).

